# Laryngeal Leech as a Live Foreign Body: Case Reports and a Safe Removal Technique

**DOI:** 10.1155/crot/6543201

**Published:** 2026-04-22

**Authors:** Mahdieh Mohebbi, Ali Ghasemi, Saeed Sohrabpour, Reza Erfanian, Farrokh Heidari, Dina Mousavi-Asl, Benyamin Mousavi-Asl

**Affiliations:** ^1^ Otorhinolaryngology Research Center, Tehran University of Medical Sciences, Tehran, Iran, tums.ac.ir; ^2^ Department of Anesthesiology, Amir A’lam Hospital, Tehran, Iran

**Keywords:** airway foreign body, larynx, leech infestation

## Abstract

Leech infestation in the larynx is a rare condition with serious implications due to airway obstruction and persistent bleeding. This report presents two cases of dysphonia and hemoptysis caused by laryngeal leeches after consuming contaminated water. Both patients underwent successful removal under general anesthesia using rigid laryngoscopy and 10% lidocaine spray for safe detachment. The cases highlight the importance of prompt diagnosis and effective techniques for managing this rare but potentially hazardous condition.

## 1. Introduction

Leeches, blood‐sucking​ parasites, are primarily found in untreated rural water. They are composed of 34 body segments and possess two suckers: a smaller anterior sucker with cartilaginous jaws for bloodsucking and a larger posterior sucker for locomotion and attachment. Leech infestation can cause severe complications in humans and animals [1].

Internal hirudiniasis is an uncommon parasitic infestation caused by leeches entering the human body through ingestion of contaminated water or swimming in infested sources. While nasal and pharyngeal attachments are more common, laryngeal involvement is rare but potentially life‐threatening due to the risk of airway obstruction and bleeding. Leeches secrete anticoagulants such as hirudin and vasodilators that prolong bleeding and may result in hemoptysis, anemia, or even acute airway compromise [2–4]. Prompt recognition and safe removal are therefore essential.

### 1.1. Case 1

A 50‐year‐old man presented with dysphonia and hemoptysis 10 days after drinking contaminated water. Initial video laryngoscopy (Figure 1(a)) at an outpatient center revealed a living foreign body, suspected to be a leech, attached to the anterior commissure through its large posterior sucker. Worsening symptoms prompted referral to our center after 2 days, where emergency surgical removal was performed.

FIGURE 1Video laryngoscopy view of the two cases of leeches in larynx. (a) Case 1: leech attached to the anterior commissure. (b) Case 2: leech attached to the epiglottis extending toward the glottis.

**Figure:**
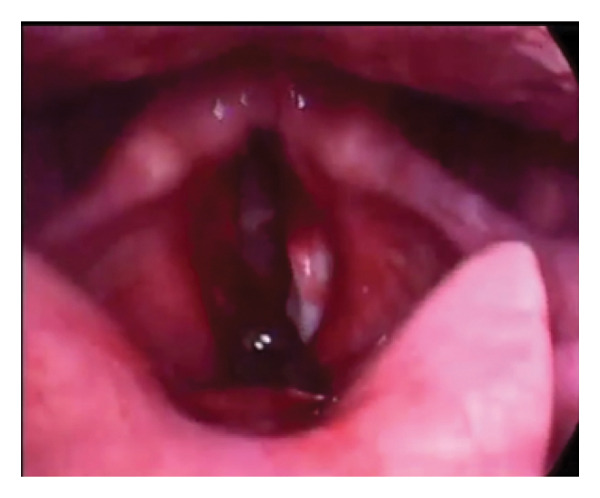
(a)

**Figure:**
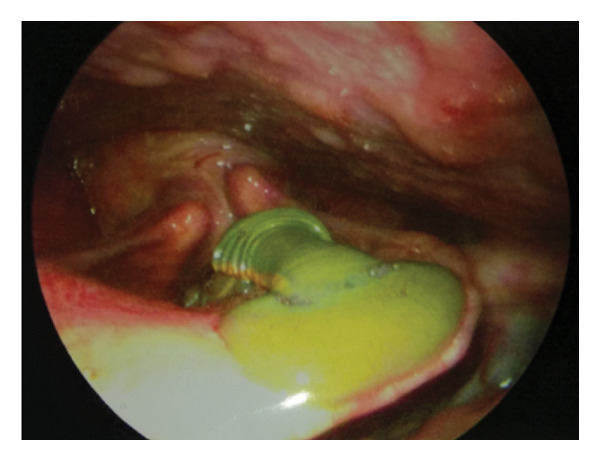
(b)

### 1.2. Case 2

A 37‐year‐old man developed dysphonia five days after drinking spring water. Video laryngoscopy at an outpatient clinic identified a living foreign body attached to the epiglottis and hanging toward the glottis (Figure 1(b)). He was referred to our tertiary center for further management.

## 2. Leech Removal

**FIGURE 2:**
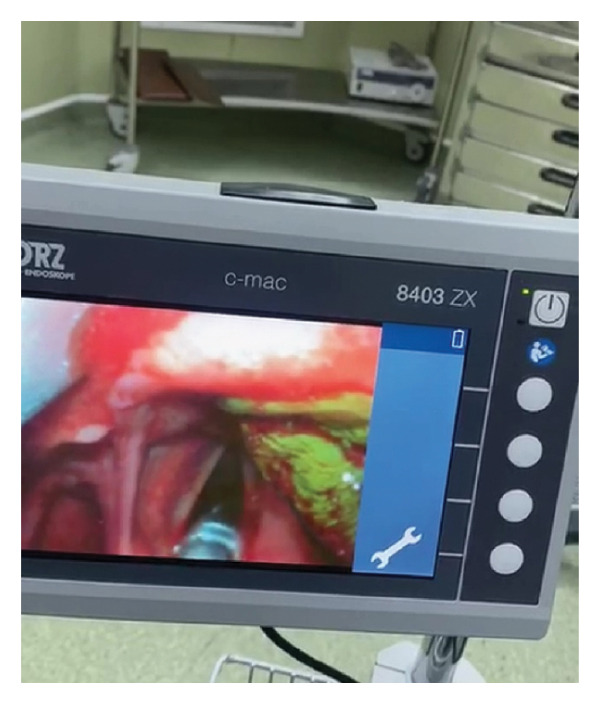
Intubation using a GlideScope video laryngoscope with a size 5 cuffed orotracheal tube prior to leech removal.

Both patients underwent emergency surgery. After anesthesia induction, gentle intubation was performed with a small cuffed orotracheal tube (size 5) using a GlideScope video laryngoscope (Figure 2). Rigid laryngoscopy exposed the leech attachment. Under microscopic magnification, the posterior sucker’s attachment was identified while the anterior was moving around.

To prevent displacement after paralysis, the leech was gently grasped using blunt forceps and sprayed with two puffs of 10% lidocaine. Within 2 min, the leech was paralyzed and safely removed after posterior attachment sucker was released. Care was taken to avoid rupture, as residual parts could cause bleeding due to hirudin in the suckers (Figure 3).

FIGURE 3Removal of the leech using blunt forceps after topical application of 10% lidocaine spray. The intact leech measured 32 mm in Case 1 (a) and 72 mm in Case 2 (b).

**Figure:**
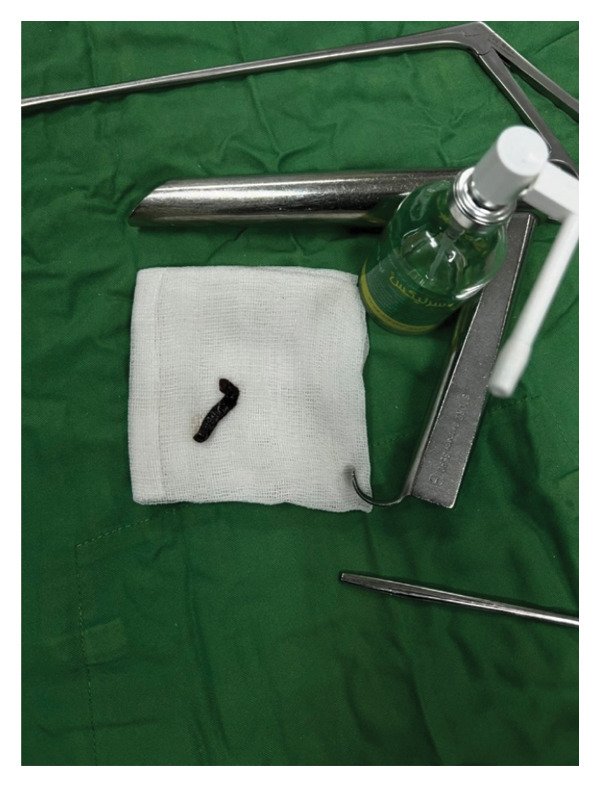
(a)

**Figure:**
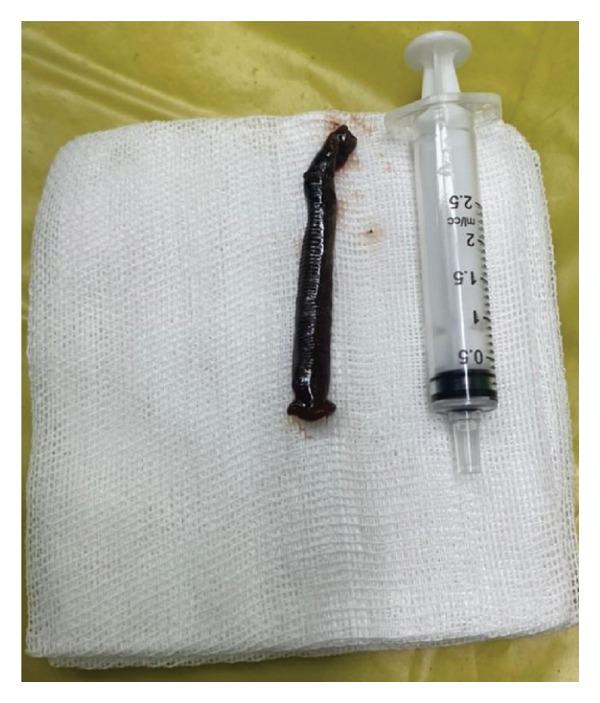
(b)

## 3. Discussion

Leech infestation in the larynx is rarely reported, mainly in Mediterranean, African, and Asian countries. Their ability to adhere to mucosa and consume blood without causing pain delays diagnosis. Continuous blood feeding combined with secretion of anticoagulants such as hirudin and vasodilatory substances can lead to persistent bleeding manifestations including hemoptysis, hematemesis, epistaxis, and chronic anemia. In laryngeal involvement, progressive enlargement of the leech during feeding may precipitate stridor, acute airway obstruction, and respiratory distress, necessitating emergent intervention [1, 5].

Various methods have been described by different authors. Extracting the leech under local anesthesia [6] could be uncomfortable for the patients. General anesthesia is the preferred approach for safe and controlled removal [7]. Inhalational anesthetics such as sevoflurane may induce paralysis, but uncontrolled detachment carries the risk of distal airway migration [3, 8, 9]. We preferred gentle intubation with a small cuffed orotracheal tube.

Several chemical agents such as hypertonic saline, adrenaline, cocaine, and strong saline irrigation have been used to facilitate detachment. However, application of these substances in the respiratory tract must be performed with extreme caution due to the risk of bronchospasm, mucosal injury, aspiration, and even suffocation. In these cases, careful intubation with a GlideScope video laryngoscope and the use of 10% lidocaine spray ensured smooth and complete removal. Lidocaine effectively paralyzes leeches, allowing safe detachment without trauma [2, 10, 11].

Advanced extraction techniques have also been described. Li and Sun reported successful removal of a leech in one piece using intrabronchial cryotherapy under local anesthesia. Although effective, such approaches require specialized equipment and technical expertise, which may not be readily available in emergency settings [12].

This method minimizes complications, including incomplete removal or persistent bleeding, and emphasizes the importance of early diagnosis and effective surgical intervention. Public health efforts to ensure access to clean water remain critical in preventing such infestations.

## 4. Conclusion

Laryngeal leech infestation is rare but potentially dangerous. Early recognition and prompt removal under general anesthesia are essential to prevent complications. The use of 10% lidocaine spray is a safe and effective method for leech detachment. These cases underscore the importance of clinician awareness, especially in endemic regions.

## Funding

No funding was received for this manuscript.

## Ethics Statement

Ethical approval was waived by the Ethics Committee of Tehran University of Medical Sciences, as this study represents a descriptive case report without experimental intervention. Written informed consent was obtained from both patients for publication of this case report and the accompanying images.

## Conflicts of Interest

The authors declare no conflicts of interest.

## Data Availability

The data that support the findings of this study are available on request from the corresponding author. The data are not publicly available due to privacy or ethical restrictions.
